# Mind matters: A narrative review on affective state-dependency in non-invasive brain stimulation

**DOI:** 10.1016/j.ijchp.2023.100378

**Published:** 2023-02-16

**Authors:** Dennis J.L.G. Schutter, Fenne Smits, Jana Klaus

**Affiliations:** a Department of Experimental Psychology, Helmholtz Institute, Utrecht University, Utrecht, The Netherlands; b Brain Research & Innovation Centre, Ministry of Defence, Utrecht, the Netherlands; c Department of Psychiatry, UMC Utrecht Brain Center, University Medical Center Utrecht, The Netherlands

**Keywords:** Embodiment, Emotion, Individual differences, Psychological state, Non-invasive brain stimulation, NIBS, non-invasive brain stimulation, TMS, transcranial magnetic stimulation, tES, transcranial electric stimulation, tDCS, transcranial direct current stimulation

## Abstract

Variability in findings related to non-invasive brain stimulation (NIBS) have increasingly been described as a result of differences in neurophysiological state. Additionally, there is some evidence suggesting that individual differences in psychological states may correlate with the magnitude and directionality of effects of NIBS on the neural and behavioural level. In this narrative review, it is proposed that the assessment of baseline affective states can quantify non-reductive properties which are not readily accessible to neuroscientific methods. Particularly, affective-related states are theorized to correlate with physiological, behavioural and phenomenological effects of NIBS. While further systematic research is needed, baseline psychological states are suggested to provide a complementary cost-effective source of information for understanding variability in NIBS outcomes. Implementing measures of psychological state may potentially contribute to increasing the sensitivity and specificity of results in experimental and clinical NIBS studies.

## Introduction

Transcranial magnetic (TMS) and electric stimulation (tES) techniques are non-invasive methods to probe and modulate brain physiology, allowing for causal inferences between neural activity and behaviour. Owing to its unique ability to influence nerve tissue, non-invasive brain stimulation (NIBS) provides an imperative means to study the workings of the human brain. In addition to investigating the basic neural properties underlying mental processes and behaviour, the modulatory effects of NIBS on neurophysiology that can outlast the stimulation period make tES and TMS suitable candidates for the treatment of mental and neurological disorders. NIBS has proven invaluable for expanding our knowledge about the neurobiological underpinnings of human behaviour in health and disease, yet fluctuations in, for example, ongoing neural activity can greatly influence the outcomes of NIBS on brain and behaviour (e.g., Feurra et al., 2013; Schutter & Hortensius, 2011). Within this context, the concept of *neural* state-dependency has been proposed, in which the effects caused by NIBS at least in part depend on ‘types’ of brain activity at the time of stimulation (Kasten & Herrmann, 2022). Acknowledging variability of physiological states within and across individuals may contribute to a better understanding of how NIBS interacts with brain matter. Moreover, it provides information on how accounting for individual differences in neural state may increase reliability and optimise the effects of NIBS (Bradley, Nydam, Dux & Mattingley, 2022).

Variation in neural activity due to external (e.g., task demands, time of day) or internal (e.g., spontaneous electroencephalography [EEG] fluctuations) factors has been demonstrated to predict magnitude and directionality of the effects of NIBS on brain physiology and behaviour (Bradley et al., 2022; Hartwigsen & Silvanto, 2022; Penton, Catmur, Banissy, Bird & Walsh, 2022). Information that can be extracted from the brain with conventional neuroimaging techniques, such as functional magnetic resonance imaging (fMRI) and EEG, is an important source for studying the brain-NIBS coupling in humans. While the biological-centred approach is critical, these techniques are restricted by physical and spatiotemporal constraints and can only focus on a limited number of neural features. Identifying response markers for neuromodulation treatment of psychiatric disorders has become an important topic in the field of NIBS, but the extent to which these markers can reliably predict behavioural outcomes to NIBS-based interventions is still part of ongoing neuroscientific research.

An additional source of information that may be relevant is one's *psychological* state. The added value of measuring psychological states is proposed to lie in (1) providing unique information, such as the phenomenological (subjective) experience of anxiety that cannot be disclosed with existing neuroimaging techniques, and (2) not having to rely on expensive neuroimaging equipment and expertise. Within the psychological framework of embodiment, psychological states can be understood as subjective phenomenological read-outs determined by sensory signals from the external surroundings (exteroception), (somato-visceral) signals from within the body (interoception), and prior experience (Northoff, 2012; Oosterwijk et al., 2012). One category of psychological states are affective states or the conscious experience of feeling the underlying emotion (Panksepp & Biven, 2012). Affective states can be either focused, short-lived sensations or more diffuse experiences that extend over time. The former would be more closely related to emotions (e.g., anxiety), whereas the latter is more associated with mood (e.g., depression) and personality characteristics (e.g., neurotic). In addition, motivational dispositions based on, for example, reward and punishment sensitivity may reflect the more latent aspects of affective states. Affective states may provide a non-reductive proxy for an individual's overall (bodily) state. It is proposed that such states can be informative for understanding and predicting the effects of NIBS on brain and behaviour both on the population and the individual level.

**Fig. 1:**
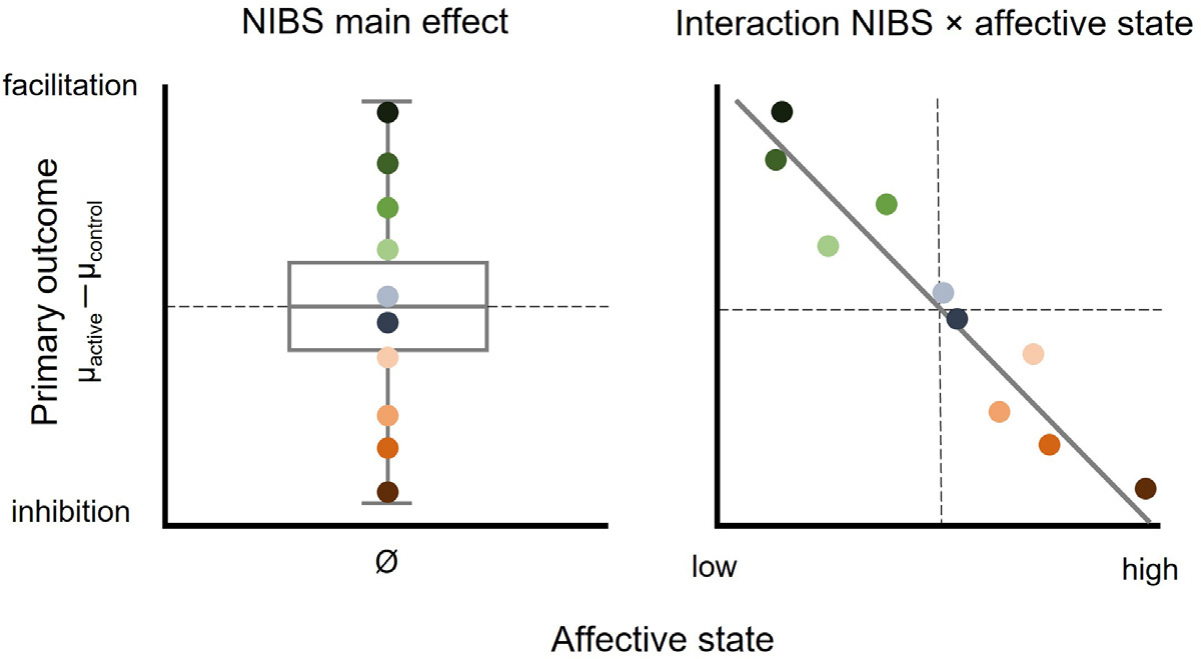
Illustration of the association between affective state and NIBS-induced effects. When not accounting for individual differences in affective state (left panel), effects from participants showing inhibition from active NIBS relative to a control condition (green shaded points) and from participants showing facilitation (red shaded points) cancel each other out, resulting in a null effect of NIBS on the group level. Individual differences in affective state may account for differences in polarity and magnitude of the NIBS effect (right panel): Individuals scoring low on an affective measure show NIBS-induced inhibition, which flips towards facilitation with increasing affective state scores. Note that the directionality of effects is hypothetical and the association can also be positive or non-linear.

The aim of this theoretical perspective is to illustrate the potential role of affective states in NIBS by reviewing studies that provide evidence for associations between baseline affective states and the magnitude and directionality of tES- and TMS-related effects on brain and behaviour. It is proposed that heterogeneity of results and null findings of NIBS studies on the group level may at least in part be accounted for by individual differences in affective states (Fig. 1).

## Affective state-dependency in TMS research

Ever since the pioneering research demonstrating that manipulating the state of neural excitability can induce opposite behavioural effects of TMS (for a review see Silvanto & Pascual-Leone, 2008), neuroscientific research has yielded important insights into the role of neural state-dependency in basic and clinical research (e.g., Borgomaneri et al., 2020; Janssens & Sack, 2021; Siebner et al., 2022; Wendt et al., 2022). Next to physiological measures, however, there is an increasing number of studies showing that psychological measures depicting individuals’ affective states prior to or during TMS can substantially contribute to observed outcomes as well (Table 1). Increases in cortical excitability during the processing of threat-related stimuli have, for instance, been repeatedly shown in healthy volunteers (Coombes et al., 2009; Hortensius, de Gelder, & Schutter, 2016; Schutter, Hofman & Honk, 2008). Moreover, anticipatory anxiety was shown to have a facilitating effect on cortical excitability in response to a suprathreshold TMS pulse (Oathes, Bruce & Nitschke, 2008), providing further evidence for relations between affective states, including worrying, nervousness, action preparedness, and cortical excitability levels.

**Table 1:** Overview of reviewed studies examining the interaction between TMS effects and affective states.

Study	Sample	TMS details	Outcome variable(s)	Affective state measurement	Finding
Baeken et al. (2011)	*N* = 24 (all female); healthy volunteers; age 19–29 years	10-Hz offline TMS, 1560 pulses, right DLPFC or sham, 110% rMT	Salivary cortisol, Mood (POMS)	STAI-state	Higher state anxiety scores associated with higher cortisol increase following active right DLPFC TMS; no significant TMS effects on mood
Berlim et al. (2013)	*N* = 14 (7 M/8F); MDD patients; age: 33–61 years	10-Hz offline TMS, 3000 pulses, 120% rMT, left DLPFC, 20 sessions	Depressive symptoms (HAM-D21)	Big Five Inventory: Extraversion and Neuroticism personality domains	Higher baseline Extraversion scores associated with more depressive symptom improvement; no effect of baseline Neuroticism scores
Chen et al. (2020)	*N* = 97 (20F/77 M); MUD patients; age >18 years	Active or sham iTBS over the left DLPFC; 20 daily sessions over 4 weeks; 100% rMT; 50 Hz, burst frequency 5 Hz, 900 pulses spread over five minutes; train duration: 2 s, intertrain-interval: 6 s; between-participant	VAS for spontaneous and cue-induced craving	PHQ-9, GAD-7	Milder anxiety and depressive symptoms correlated with reduced craving in response to active iTBS vs. sham
De Witte et al. (2020)	*N* = 38 (all female); *M*_age_ = 23.5, *SD* = 3.0	Active or sham iTBS over the left DLPFC; 110% rMT; 50 Hz, burst frequency 5 Hz, 1620 pulses spread over 54 cycles (10 bursts of 3 pulses each); train duration: 2 s, inter-train interval: 6 s; within-participant	Trait rumination measured by Ruminative Response Scale (reflective pondering and depressive brooding); momentary rumination measured by Ruminative Self-Focus Scale before TSST and after TBS; cortisol levels 15 and 25 min after TSST	TSST	Under sham cTBS, rumination increased with increasing brooding levels, but this effect was not observed under active cTBS Higher levels of brooding related to decreased cortisol secretion under active, but not sham cTBS
Downar et al. (2014)	*N* = 47 (20 M/27F); patients with unipolar (*N* = 38) or bipolar depression (*N* = 9)	10-Hz offline TMS, 3000 pulses, 120% rMT, DMPFC, 20 sessions	Depressive symptoms (HAM-D17)	BDI-II, QIDS	Higher baseline anhedonia symptoms (BDI-II items ‘Pessimism’ and ‘Loss of Pleasure’, QIDS item ‘General Interest’) associated with lower symptom improvement
Guo et al. (2020)	*N* = 44 (24F/20 M); healthy volunteers; *M*_age_ = 21, *SEM* = 0.39	Single-pulse TMS 90, 100, 110, 120, 130, 140 or 150 ms after stimulus onset, or no TMS over early visual cortex at 120% phosphene detection threshold	Emotion detection task (angry vs. happy vs. fearful faces)	STAI, BAI	Higher TMS-induced disruption of anger recognition for individuals with lower anxiety levels; no modulation of fear or happiness recognition
Sagliano et al. (2016)	*N* = 22 (all female); healthy volunteers; age: 19–30 years	Single-pulse TMS 100 or 200 ms after stimulus onset to left or right DLPFC or sham vertex	Attentional bias for threatening pictures (exogenous cueing task)	STAI-trait	Higher trait anxiety associated with higher disengagement bias during left DLPFC stimulation
Vanderhasselt et al. (2011)	*N* = 28 (all female); healthy volunteers; age: 18–29 years	10-Hz offline TMS, 1560 pulses, right DLPFC or sham	Attentional bias for angry faces (exogenous cueing task)	POMS, STAI-state	Higher state anxiety scores associated with increased attentional bias for angry faces following active right DLPFC TMS

BDI-II = Beck Depression Inventory-II; cTBS = continuous Theta Burst Stimulation; DLPFC: Dorsolateral Prefrontal Cortex; DMPFC: Dorsomedial Prefrontal Cortex; F = Females; GAD-7 = Generalized Anxiety Disorder-7; HAM-D17/HAM-D21 = ​​17/21-item Hamilton Depression Rating Scale; iTBS = intermittent Theta Burst Stimulation; M = Males; *M* = Mean; MDD = Major Depressive Disorder; MEP = Motor-Evoked Potential; MUD = Methamphetamine Use Disorder; NEO-PI-*R* = NEO Personality Inventory Revised; PANAS = Positive and Negative Affect Schedule; PEMS = Palatable Eating Motives Scale; PHQ-9: Patient Health Questionnare-9; POMS = Profile of Mood States; QIDS = Quick Inventory of Depressive Symptomatology–Self-rated 16-item scale; rMT = resting Motor Threshold; *SD* = Standard Deviation; SEM = Standard Error of the Mean; STAI = State-Trait Anxiety Inventory; TMS = Transcranial Magnetic Stimulation; TSST = Trier Social Stress Test; VAS = Visual Analogue Scale.

Additionally, affective state-dependent modulation of NIBS effects have been reported for different outcome measures. A sham-controlled study tested the influence of state anxiety on the endocrinological response to a single session of high-frequency repetitive TMS (rTMS) over the right dorsolateral prefrontal cortex (DLPFC) in healthy female volunteers (Baeken, Vanderhasselt & De Raedt, 2011). Results showed that individuals with higher state anxiety displayed significantly higher levels of the stress hormone cortisol after real TMS as compared to those scoring low on state anxiety. The authors proposed that the inclusion of individual anxiety states in experimental rTMS research could lead to a deeper understanding of the effects of NIBS on the brain's stress system (Baeken et al., 2011). Another study examined the effects of state anxiety prior to a session of high- frequency rTMS to the right DLPFC on attention processing in healthy volunteers (Vanderhasselt, Baeken, Hendricks & De Raedt, 2011). Self-report measures of state anxiety prior to stimulation correlated positively with behavioural performance. More specifically, participants with higher state anxiety showed a more pronounced increase of attentional bias towards negative information after high-frequency rTMS over the right DLPFC. Interference in the prefrontal-amygdala pathway was speculated to provide a possible neural basis for the findings (Vanderhasselt et al., 2011). Indeed, the close PFC-amygdala link to the brain's stress axis offers a neurobiological framework for unifying the cortisol findings by Baeken et al. (2011) and the effects on attentional bias reported by Vanderhasselt et al. (2011). In another attentional bias study, the lateralized role of the left and right DLPFC in early threat processing was examined in low- and high-anxious participants using a single-pulse online TMS protocol to interfere with cortical processing (Sagliano, D'Olimpio, Panico, Gagliardi & Trojano, 2016). Here, left DLPFC interference in high-anxious participants increased individual attentional bias to threat cues. By contrast, low-anxious participants showed an attentional bias away from threat cues in response to TMS-induced interference with left-sided cortical processing (Sagliano et al., 2016). Similarly, Guo, Calver, Soornack and Bourke (2020) found that single-pulse TMS to the visual cortex disrupted emotion recognition of angry faces more in low-trait anxiety participants than in high-trait anxiety participants.

Research in clinical settings also provides evidence for affective state-dependent effects. For example, an open-label adjuvant study investigated the antidepressant effects of bilateral high-frequency rTMS of the prefrontal cortex in medication-resistant patients with a major depressive episode (Downar et al., 2014). Higher levels of baseline anhedonia (e.g., loss of pleasure and interest) were observed in patients who failed to respond to rTMS as compared to patients who showed an antidepressant effect. Moreover, higher anhedonia levels were associated with reduced resting-state functional connectivity in the brain's reward circuit, suggesting a potential neurobiological link between anhedonic state and antidepressant response to rTMS. Berlim, McGirr, Beaulieu, Van den Eynde and Turecki (2013) reported a positive association between extraversion and treatment outcome of high-frequency TMS in major depressive disorder (MDD) patients, while neuroticism had no influence. In another study, the role of baseline anxiety, depression and impulsivity on the efficacy of adjuvant intermittent theta burst stimulation (iTBS) treatment to reduce craving was explored in a group of patients with methamphetamine use disorder (Chen et al., 2020). Lower levels of anxiety and depression and higher levels of non-planning impulsivity were associated with a higher probability of a positive treatment outcome to iTBS. Interestingly, this finding fits results showing that the effects of iTBS on the speed of cortisol response recovery after the Trier Social Stress Test depend on the individual tendency to use the maladaptive rumination style ‘depressive brooding’ (De Witte et al., 2020). Altogether, these studies suggest that the interactions between affective state and TMS can be informative about research in healthy volunteers as well as treatment of patients.

## Affective state-dependency in tES research

Following the empirical confirmation that weak electric currents applied to the scalp induce changes in cortical excitability (Nitsche & Paulus, 2000), transcranial direct current stimulation (tDCS) has become a widely used technique to modulate brain physiology and behaviour in healthy volunteers. Despite its significant value to brain research, large individual differences in responsivity have been observed. These can in part be explained by, for instance, variation in scalp-to-cortex distances, neuro-anatomical features (e.g., gyral folding), electrode montages, and physiological states (Bradley et al., 2022; Opitz, Paulus, Will, Antunes & Thielscher, 2015). However, the role of affective state on the effects of tDCS provides another point of entry for accounting for intra- and interindividual variability. Evidence for such an association comes from studies that explored the effects of tDCS on motivation and emotion (Table 2).

**Table 2:** Overview of reviewed studies examining the interaction between tES effects and affective states.

Study	Sample	tES details	Outcome variable(s)	Affective state measurement	Finding
Abend et al. (2019)	*N* = 16 (7F/9 M); healthy volunteers; *M*_age_ = 25.6, *SD* = 2.5	tDCS; 15 min, 1.5 mA; online; anode centrally over forehead, cathode below inion; active or sham; within-subjects design	Response to emotional and neutral video clips	BDI-II	Stronger tDCS-induced increase in sgACC activity during negative video clips associated with higher baseline depressive symptoms
Ankri et al. (2020)	*N* = 69 (all female); healthy volunteers; *M*_age_=24, *SD*=2	tDCS, 20 min, 2 mA, anode placed right frontal (F4-AF4)/cathode placed on Cz, or sham	Working memory performance (n-back task)	High stress condition (TSST) vs. low stress condition (control)	Reduced accuracy following tDCS in high vs. low stress condition
Esposito et al. (2022)	*N* = 18 (8 M/10F); healthy volunteers; *M*_age_ = 24, *SD* = 4	tDCS, 17 min, 1 mA, anode placed left frontal (F3)/cathode placed on right supraorbital area, or sham	Reaction times (RT) and pupil dilation in auditory oddball task	STAI-state	Higher state anxiety scores and reduced pupillary response associated with slower RT following tDCS
Gilam et al. (2018)	*N* = 25 (15F/10 M); healthy volunteers; *M*_age_ = 26.16, *SD =*3.63	1.5 mA anodal tDCS, 22 min, offline (during provocation); anode over central forehead, cathode on right shoulder; active or sham; within-subjects design	Aggressive behaviour towards fictional opponent in TAP following anger-infused ultimatum game (aiUG)	Emotion regulation strategies: Trait suppression and trait reappraisal	Higher tendency for emotion suppression associated with larger tDCS-induced reduction in anger following provocation; no effect of participants who reported a stronger tendency to use suppression as an emotion regulation strategy, showed a greater effect of reappraisal
Hortensius et al. (2012)	*N* = 60; healthy volunteers; no age and sex distributions reported	2 mA tDCS; 15 min offline; bilateral DLPFC (F3/F4 or F4/F3) or sham between participants	Aggressive behaviour towards fictional opponent in TAP, following interpersonal insults	Self-reported anger (following insult minus baseline)	Increased aggression in high-anger participants receiving anodal tDCS to the left DLPFC
Peña-Gómez et al. (2011)	*N* = 16 (all female); healthy volunteers; *Mean*_age_= 22.93, *SD* = 4.18	tDCS, 20 min, 1 mA; anode over left PFC (F3), cathode over right motor cortex (C4), or sham; within-subjects design	Valence rating of IAPS pictures (positive, negative, neutral)	Before and after tDCS: VAS (nervousness, contentment, sadness, hope and annoyance); before tDCS: PANAS, STAI-state, NEO-FFI	Negative correlation between extraversion and reduced negative ratings of negative (but not positive or neutral) pictures
Rabipour et al. (2018)	single-blind: *N* = 52 (31F/21 M), *M*_age_ = 20.5, *SD* = 1.9; double-blind: *N* = 38 (22F/16 M), *M*_age_ = 20.6, *SD* = 3.4	tDCS, 20 min, 2 mA; online; anode over F3, cathode over right supraorbital area; active or sham, between-subjects design	N-back working memory task	Expectation priming: High (i.e., tDCS is effective in improving performance) vs. low (i.e., no known benefits of tDCS)	Better performance under active tDCS following high vs. low expectation priming
Rabipour, Vidjen, Remaud, Davidson, & Tremblay, 2019	*N* = 121 (88F/33 M); healthy volunteers; *M*_age_ = 21.1, *SD* = 3.6	tDCS, 20 min, 2 mA; anode over left or right motor cortex (corresponding to preferred or non-preferred hand, respectively), cathode over contralateral supraorbital region; active or sham; between-subjects design	Grooved Pegboard Test	Expectation priming: High (i.e., tDCS is effective in improving performance) vs. low (i.e., no known benefits of tDCS)	No modulation of motor performance as a function of tDCS or expectation priming
Ray et al. (2017)	*N* = 18 (10F/8 M) obese volunteers; *M*_age_ = 22.7, *SD* = 7.9	tDCS, 20 min, 2 mA; cathode over F3, anode over F4; active or sham; within-subjects design	Food craving pre- and post-tDCS; actual food intake (amount of calories eaten) post-tDCS	BIS-11, DEBQ-R	Lower food craving following active tDCS in females scoring lower on attentional impulsivity; lower preferred-food intake in males not focused on calorie restriction; lower total food intake in males with higher non-planning impulsivity
Ray et al. (2019)	*N* = 74 (44F/30 M) obese volunteers; *M*_age_ = 19.9, *SD* = 3.4,	tDCS, 20 min, 2 mA; cathode over F3, anode over F4; active or sham; within-subjects design	Food craving pre- and post-tDCS; actual food intake (amount of calories eaten) post-tDCS	Manipulation of expectation of behavioural modulation of tDCS; BIS-11, DEBQ-R, PEMS, BES	Expecting positive effect of tDCS decreased food craving and caloric intake, regardless of tDCS condition (active/sham); no effect of questionnaire scores
Riddle et al. (2022)	*N* = 82 (66F/16 M); MDD patients (*N* = 41) and healthy volunteers (*N* = 41); age: 18–65 years	tACS at individual alpha frequency, 40 min, 1 mA zero-to-peak, bifrontal (F3/F4, return electrode: Cz) or sham	Alpha power in resting-state and during emotionally salient IAPS picture presentations	BDI-II	tACS reduced the left frontal alpha power increase during resting-state and positive picture viewing in the MDD group; higher baseline depression scores were associated with a stronger reduction in alpha power following tACS
Riddle et al. (2022)	*N* = 3 (all female); PMDD patients; age not reported	10-Hz tACS, 40 min, 1 mA zero-to-peak, bifrontal (return electrode not reported)	Alpha power in resting-state	Late luteal phase (PMDD symptoms high) vs. follicular phase (PMDD symptoms low) of menstrual cycle	In the late luteal phase, tACS increased midline frontal alpha power in all three participants, while in the follicular phase, tACS had no consistent effect on alpha power
Sagliano et al. (2017)	*N* = 40 (all females); healthy volunteers; *M*_age_ = 22.95, *SEM* = 0.48	tDCS; 15 min, 1 mA; offline; bilateral prefrontal montage (F3/F4); left-anodal/right-cathodal, left-cathodal/right-anodal, or sham; within-subjects design	Exogenous cueing task with threatening and non-threatening IAPS pictures	STAI	Following right-cathodal/left-anodal tDCS, higher attentional capture by threatening stimuli for individuals scoring lower on trait anxiety, and longer attentional holding by threatening stimuli for individuals scoring higher on trait anxiety
Wang et al. (2022)	*N* = 70 (all female); healthy volunteers; *M*_age_ = 19.6, *SD* = 1.55	tDCS, 20 min, 1.5 mA; anode over right PFC (F4), cathode over left PFC (F3); active or sham; between-subjects design	Performance on AUT and RAT before and after stress induction	STAI, BDI-II, PANAS TSST before tDCS	Lower tDCS-induced performance impairment on flexibility component of AUT mediated by state anxiety; no effects of other measures

AUT = Alternative Uses Task; BAI = Beck Anxiety Inventory; BES = Binge Eating Scale; BIS-11 = Barratt Impulsiveness Scale 11; DEBQ-*R* = Dutch Eating Behaviour Questionnaire-Restraint;  F = Females; IAPS = International Affective Picture System; M = Males; *M* = Mean; MDD = Major Depressive Disorder; NEO-FFI = shortened version of NEO Personality Inventory; PANAS = Positive and Negative Affect Schedule; PEMS = Palatable Eating Motives Scale; PMDD = Premenstrual Dysphoric Disorder; PSAP = Point Subtraction Aggression Paradigm; RAT = Remote Associations Task; SD = Standard Deviation; SEM = Standard Error of the Mean; sgACC = subgenual Anterior Cingulate Cortex; STAI = State-Trait Anxiety Inventory; tACS = transcranial Alternating Current Stimulation; TAP = Taylor Aggression Paradigm; tDCS = transcranial Direct Current Stimulation; TSST = Trier Social Stress Test; VAS = Visual Analogue Scale.

In a sham-controlled study, tDCS over the frontal cortex was combined with a revised Taylor aggression paradigm in healthy volunteers (Hortensius, Schutter & Harmon-Jones, 2012). State anger was manipulated by presenting participants with insulting feedback from a fictional person. Results showed no main effect of tDCS, but when levels of induced state anger were taken into account, tDCS was found to increase the administration of noise blasts to the fictional person. Behavioural effects of tDCS on anger in the anger-infused ultimatum game have also been shown to depend on individuals’ emotion regulation style as shown by a cross-over sham-controlled double-blind study (Gilam et al., 2018). Individuals with a tendency to suppress rather than reappraise negative emotions showed decreased anger under active relative to sham tDCS.

The importance of considering individual differences is further underlined by a study showing that individuals scoring high on the introversion personality dimension are more susceptible to the modulatory effects of tDCS on emotional reactivity than individuals scoring high on extraversion (Peña-Gómez, Vidal-Piñeiro, Clemente, Pascual-Leone & Bartrés-Faz, 2011). In a more recent sham-controlled tDCS study on stress-induced creativity in healthy volunteers, mediation analysis showed that state anxiety explained more than 60% of the stress-reduced performance decrement following active as compared to sham bilateral prefrontal tDCS (Wang et al., 2022). The modulation of neural excitability of the DLPFC was speculated to allow for more effective top-down regulation of the subcortical brain regions associated with anxiety. Levels of self-reported depression have also been found to predict the effects of tDCS on behaviour (Abend et al., 2019). Compared to sham tDCS, active fronto-cerebellar tDCS reduced perceived negative emotions in an emotion induction task in healthy volunteers. Importantly, individual depression scores during sham tDCS were negatively correlated with the neural response in the subgenual anterior cingulate cortex, while this pattern was reversed during active tDCS. This suggests that individual differences in depressive mood can moderate the effect of tDCS during emotion regulation, with differences becoming more pronounced in individuals with high levels of depressive mood. Moreover, Sagliano, D'Olimpio, Izzo and Trojano (2017) showed that anodal tDCS to the right DLPFC increased attentional bias towards threatening stimuli in high-anxious women, while it increased attentional capture by threatening stimuli in low-anxious women. Recently, Esposito, Ferrari, Fracassi, Miniussi and Brignani (2022) found that anodal tDCS over the left prefrontal cortex improved reaction times in an auditory oddball task relative to baseline performance in healthy volunteers with low self-reported state anxiety. By contrast, individuals with higher state anxiety performed significantly worse under anodal tDCS relative to baseline performance. These findings were corroborated by concomitant pupil dilation measures, leading the authors to emphasise the crucial role of taking baseline arousal into account when anticipating tDCS-induced performance changes. Moreover, it has been shown that administering tDCS over the right prefrontal cortex can improve working memory performance in no-stress conditions, while under stress (Trier Social Stress task), the application of tDCS can impair performance (Ankri, Braw, Luboshits & Meiron, 2020).

In clinical populations, there is increasing evidence for associations between affective state and NIBS-induced effects as well. A study on alpha hyperactivity in MDD patients (Riddle, Alexander, Schiller, Rubinow & Frohlich, 2022) showed that transcranial alternating current stimulation (tACS) at the individual alpha peak frequency (8–12 Hz) attenuated left frontal alpha hyperactivity to a significantly stronger extent in patients with higher MDD symptom severity at baseline. The state-dependency of this effect was later replicated in a case series with three women with premenstrual dysphoric disorder, who showed a significant increase in frontal alpha hypoactivity when alpha-tACS was applied in the luteal phase compared to the follicular phase of their menstrual cycle, for example, when symptom severity is highest (Riddle, Rubinow and Frohlich, 2022). In another study on obesity and food intake using a food photo craving task, the effects of bipolar tDCS of the DLPFC depended on sex and one's ability to control impulses (Ray et al., 2017). More specifically, tDCS significantly reduced food craving in obese females with lower attentional impulsivity as assessed with the self-report Barratt Impulsiveness Scale. By contrast, for obese males, tDCS significantly reduced food intake in individuals with higher non-planning impulsivity scores. Despite the small sample size (10 female and 8 male participants), these findings suggest an association between sex and trait impulsivity on the magnitude of the tDCS response. In a between-participant design, a follow-up study investigated to what extent expectation priming of the tDCS effect modulated obesity-related eating and craving patterns (Ray et al., 2019). While all volunteers were told at the beginning of the study that active tDCS had been shown to reduce craving and caloric intake, the participant-specific instruction (i.e., whether they would receive active or sham tDCS) as well as the actual stimulation administered (active or sham) were manipulated. Results showed no tDCS-induced effect, but participants being told to receive beneficial tDCS (regardless of the actual stimulation condition) reported craving reduced by about 10% and caloric intake by almost 40%. These findings suggest that prior subjective expectations of the efficacy of tDCS are a powerful psychological mechanism potentially obscuring any (weak) stimulation effect. However, Rabipour, Wu, Davidson and Iacoboni (2018) found an interaction of expectation priming (i.e., being told that tDCS improves or impairs performance, respectively) and actual stimulation (active or sham prefrontal tDCS) on performance in an n-back task. That is, individuals receiving active tDCS who were told that tDCS had a negative effect on memory performance indeed performed worse than participants receiving active tDCS who were primed positively. Interestingly, these findings could not be replicated with tDCS over bilateral motor cortex aimed at improving motor performance (Rabipour, Vidjen, Remaud, Davidson, & Tremblay, 2019), suggesting the importance of context in expectation-related effects. Collectively, these findings suggest that aside from individual differences in state and trait emotions, subjective expectations of the effects of stimulation may influence the effect of NIBS on behaviour. Crucially, the mentioned studies targeted different domains and brain regions, and the extent to which positive expectations influence the effects of NIBS on performance warrants further research. Nonetheless, assessing or even actively manipulating individuals’ expectations on the efficacy of the stimulation holds promise of augmenting more subtle NIBS effects.

## Linking affective state to neural excitability

Affective state-dependency of NIBS may at least in part be explained by the link between affective state and cortical excitability, which influences the susceptibility to TMS and tES. Cortical excitability can be indexed by recording the motor evoked potential (MEP) from different finger muscles following a suprathreshold TMS pulse administered over the motor cortex. Associations between resting-state cortical excitability and affective states have been extensively studied. For example, a previous study has shown that asymmetries in left and right cortical excitability are correlated with approach and avoidance-related motivational tendencies (Schutter, De Weijer, Meuwese, Morgan & Van Honk, 2008). Moreover, anticipatory anxiety and exposure to threat-related stimuli were reported to increase cortical excitability levels as indexed by higher MEP amplitudes (Coombes et al., 2009; Hortensius, de Gelder, & Schutter, 2016; Oathes et al., 2008; Schutter et al., 2008). Importantly, as the MEP in response to single-pulse TMS applied to the motor cortex results from signal propagation along the cortico-spinal tract to the motor endplates, it represents only a proxy for cortical excitability. A more direct correlate of neural excitability can be obtained by applying so-called paired-pulse TMS. In this protocol, a conditioning TMS pulse is preceded by a suprathreshold test pulse to the primary motor cortex (for a review see Hallett, 2007). Paired-pulse TMS allows for studying intracortical inhibitory and facilitatory process underlying the MEP. Higher levels of neuroticism have been associated with increased resting-state cortical excitability caused by lower intracortical inhibition in healthy volunteers (Wassermann, Greenberg, Nguyen & Murphy, 2001). Moreover, novelty seeking/approach-motivation was negatively correlated with intracortical inhibition in social anxiety disorder patients (Pallanti et al., 2010), while higher anxiety symptom severity was linked to higher intracortical facilitation in patients with generalised anxiety disorder (Li et al., 2017). Interestingly, in patients with knee osteoarthritis, higher levels of self-reported anxiety and pain perception were correlated with *l*o*wer* intracortical facilitation and *higher* intracortical inhibition (Simis et al., 2021). In contrast to the relationship with these affective measures, the pain experienced in specific movement situations, such as walking, was associated with *higher* intracortical facilitation and *lower* intracortical inhibition. The complex pattern of relations may suggest differential contributions of affective state and situational pain to cortical excitability in these patients (Simis et al., 2021).

In sum, previous research indicates that affective states are linked to cortical excitability levels, thereby providing a psychobiological basis for interactions between affective states and NIBS interventions.

## Limitations and challenges

Different findings have been found for self-reported state and trait anxiety as assessed by Spielberger's State and Trait Anxiety Inventory (STAI). For example, Vanderhasselt et al. (2011) found associations with state anxiety, whereas Sagliano et al. (2016, 2017) observed a moderating effect of trait, but not state anxiety on NIBS-induced outcomes. This begs the question whether timepoint-specific (i.e., state) or persistent (i.e., trait) measures of individual affective states are more promising in accounting for interindividual variability in NIBS outcomes. While trait and state anxiety of the STAI are positively correlated both during baseline as well as during threatening situations (Leal, Goes, da Silva & Teixeira-Silva, 2017), there is some evidence to suggest that the trait scale of the STAI also measures depression and general negative affect (Bieling, Antony & Swinson, 1998). These findings may point towards the relevance of selecting affective measures tailored to the experimental design and research question.

Self-report questionnaires are not without shortcomings and can include response biases (e.g., social desirability), and a lack of a person's ability for introspection can have a negative impact on the reliability and validity of the measurement. Differences in construct validity of self-report scales and questionnaires as well as contextual factors like subject's expectations and motivation to participate could therefore potentially explain inconsistencies observed in the literature so far. Higher levels of anxiety have, for instance, been associated both with increased and decreased intracortical facilitation, but in the first case the association was based on the Hamilton anxiety rating scale in generalized anxiety patients (Li et al., 2017) and in the latter case on the Hospital Anxiety and Depression Scale in arthritis patients (Simis et al., 2021). The relationship between affective state and NIBS outcome may seem inconsistent when different measurement instruments are used or different populations are tested. By consistently surveying both state and trait levels of affect in the context of NIBS application, future studies could assess this discrepancy and contribute to a better understanding of the driving force behind affective state-dependency.

It is important to emphasise that we do not aim to equate affective state with disease-specific predictors (e.g., symptom severity in depression to foresee treatment success). Instead, we propose that fluctuating experiences of, for example, anxiety or anger, which are specific to a given stimulation session, may be more informative to anticipate the observed NIBS-induced effect. Crucially, based on the reviewed studies, such a pattern has been observed in both clinical and non-clinical samples, suggesting that it could be a generic mechanism not restricted to specific (psycho)pathologies. Still, it needs to be stressed that assessing individual affective states to better explain NIBS effects has predominantly been done in a post-hoc manner so far. Arguably, this increases the risk of false positives. It is therefore suggested to systematically investigate the predictive value of affective states on the direction and size of NIBS effects to scrutinize whether this concept is tenable. In addition, assessing psychological states beyond cross-sectional self-reports may include longitudinal follow-ups to monitor changes in psychological states and adding behavioural correlates by administering psychological tasks may further contribute to the validity of affective states.

Furthermore, the studies reviewed here included measures of either natural fluctuations (e.g., self-reported anxiety across several time points) or experimentally induced (e.g., self-reported anxiety after a stress test) affective states (see also Tables 1 and 2). It is not unreasonable to assume that affective states caused by experimental manipulation show a different relation to the effects of NIBS as compared to affective states acquired during rest prior to NIBS. However, the extent to which these states within and across individuals differentially affect NIBS remains unclear and should be addressed empirically. Also, the vast majority of studies report findings from single experimental sessions. How ‘natural’ or NIBS-induced changes in psychological states relate to long-term effects in the treatment of disorders is an important avenue for further research. To the best of our knowledge, there is no strong evidence yet that changes in psychological states *during* multiple sessions lead to more predictable outcomes of NIBS.

Lastly, based on the limited existing evidence it is not yet viable to determine whether effects of affective state reflect, for example, arousal and/or are driven by valence (e.g., positive or negative). In light of improving the predictive value of affective state-dependency, it may be worthwhile to try to disentangle these two aspects empirically. For example, anxiety and anger are both affective states associated with increased arousal, but the specific emotional experience of the two states is different. At this point it remains unclear whether the measured affective state needs to be aligned with the study outcome measure, or whether a generic arousal measurement suffices. Gaining more insight into this association may shed more light on the link between affective states and NIBS effects prospectively. Administering validated questionnaires, such as a shortened version of the Profile of Mood States (Shacham, 1983), the Behavioural Inhibition (BIS) and Activations Scales (BAS) (Carver & White, 1994), or the State-Trait Anxiety and Anger Inventory (Spielberger, Sydeman, Owen, & Marsh, 1999) allows to quickly and easily assess the emotional and motivational aspects of affective states prior to NIBS. This way, it becomes feasible to investigate potential interactions between individual differences in affective states and effects of interventions in healthy as well as treatments in clinical populations. Similar associations have been reported in the context of other forms of treatment. For example, positive emotion scores on the center for Epidemiologic Studies Depression scale were found to correlate with higher overall functional status, including motor and cognitive performance at three-month follow-up after adjustment for relevant risk factors in stroke patients who underwent medical rehabilitation (Ostir, Berges, Ottenbacher, Clow, & Ottenbacher, 2008). In another study, baseline BAS scores were inversely correlated with the six-month course of depression (McFarland, Shankman, Tenke, Bruder, & Klein, 2006). The correlation was controlled for clinical depressive symptoms, indicating that the BAS score, as a subjective measure of dispositional tendencies associated with reward and appetitive motivation, explains unique variance and may potentially contribute to the antidepressant efficacy of NIBS. These studies suggest that the use of questionnaires may have added value in predicting responses to NIBS by taking into account individual differences in affective states.

## Conclusion

Unravelling the physiological mechanisms by which NIBS techniques establish their effects in the brain remains critical for addressing basic neuroscientific research questions and developing effective neuromodulation-based treatments. In addition to studying neural states directly, evaluation of affective states could be a cost-effective way to obtain valuable non-reductive readouts which together with neurophysiological markers offer important mediators of the effects of NIBS both in basic research as well as in applied settings. Here, empirical evidence was presented to illustrate this idea and to raise the possibility that affective states can have added value in explaining intra- and interindividual study-outcome variability. Systematic assessment of affective states in experimental and clinical research may contribute to further increasing the sensitivity and specificity of tES- and TMS-based interventions.

## Funding

This work was supported by the Dutch Research Foundation (NWO, VI.C.181.005).

## Declaration of competing interests

The authors have no competing interests to declare.
